# Cytolytic Recombinant Vesicular Stomatitis Viruses Expressing STLV-1 Receptor Specifically Eliminate STLV-1 Env-Expressing Cells in an HTLV-1 Surrogate Model In Vitro

**DOI:** 10.3390/v14040740

**Published:** 2022-03-31

**Authors:** Yohei Seki, Tomoya Kitamura, Kenta Tezuka, Megumi Murata, Hirofumi Akari, Isao Hamaguchi, Kazu Okuma

**Affiliations:** 1 Department of Safety Research on Blood and Biological Products, National Institute of Infectious Diseases, Tokyo 208-0011, Japan; yseki@niid.go.jp (Y.S.); t.kitamura@affrc.go.jp (T.K.); tezukakn@niid.go.jp (K.T.); 130hama@niid.go.jp (I.H.); 2 Exotic Disease Group, National Institute of Animal Health, National Agriculture and Food Research Organization (NARO), Tokyo 187-0022, Japan; 3 Primate Research Institute, Kyoto University, Inuyama 484-8506, Japan; mmegumi0604@gmail.com (M.M.); akari.hirofumi.5z@kyoto-u.ac.jp (H.A.); 4 Institute for Frontier Life and Medical Sciences, Kyoto University, Kyoto 606-8507, Japan; 5 Department of Microbiology, Kansai Medical University, Osaka 573-1010, Japan

**Keywords:** HTLV-1, HTLV-1 surrogate model, STLV-1, recombinant VSV, viral receptor, envelope protein

## Abstract

Human T-cell leukemia virus type 1 (HTLV-1) causes serious and intractable diseases in some carriers after infection. The elimination of infected cells is considered important to prevent this onset, but there are currently no means by which to accomplish this. We previously developed “virotherapy”, a therapeutic method that targets and kills HTLV-1-infected cells using a cytolytic recombinant vesicular stomatitis virus (rVSV). Infection with rVSV expressing an HTLV-1 primary receptor elicits therapeutic effects on HTLV-1-infected envelope protein (Env)-expressing cells in vitro and in vivo. Simian T-cell leukemia virus type 1 (STLV-1) is closely related genetically to HTLV-1, and STLV-1-infected Japanese macaques (JMs) are considered a useful HTLV-1 surrogate, non-human primate model in vivo. Here, we performed an in vitro drug evaluation of rVSVs against STLV-1 as a preclinical study. We generated novel rVSVs encoding the STLV-1 primary receptor, simian glucose transporter 1 (JM GLUT1), with or without an AcGFP reporter gene. Our data demonstrate that these rVSVs specifically and efficiently infected/eliminated the STLV-1 Env-expressing cells in vitro. These results indicate that rVSVs carrying the STLV-1 receptor could be an excellent candidate for unique anti-STLV-1 virotherapy; therefore, such antivirals can now be applied to STLV-1-infected JMs to determine their therapeutic usefulness in vivo.

## 1. Introduction

Human T-cell leukemia virus type 1 (HTLV-1) is a human pathogenic retrovirus that causes serious and intractable diseases, such as adult T-cell leukemia (ATL) and HTLV-1-associated myelopathy/tropical spastic paraparesis (HAM/TSP) [1,2,3,4,5,6]. HTLV-1 infects CD4 lymphocytes mostly through breastfeeding in infancy or sexual intercourse in adults, or rarely, through the placenta [7,8,9]. It is estimated that there are currently 10–20 million HTLV-1-infected individuals worldwide [10]. HTLV-1 infection is endemic in southern Japan, the Caribbean, South America, the Solomon Islands, and West and Central Africa [10,11]. In Japan, an estimated 1 million people, corresponding to 1% of the total population, are thought to be HTLV-1 carriers [10,12]. Additionally, it is estimated that more than 4000 horizontal new infections occur annually in Japan [13]. Although most HTLV-1 carriers remain asymptomatic for life, part of them will develop either ATL or HAM/TSP [14,15,16,17,18]. Several anti-ATL therapies are available, including chemotherapy, allogeneic hematopoietic stem cell transplantation (HSCT) [19], and anti-CCR4 antibody therapy [20]; however, ATL is frequently resistant to all these therapies, and its prognosis remains poor [21,22]. For example, the median overall survival for patients receiving chemotherapy is approximately 13 months [23]. In addition, HSCT, the only curative therapy, is performed for patients with the indication (e.g., whether there are suitable human leukocyte antigen (HLA)-matched donors, and conditions such as recipient’s age, physical strength, and organ functions), but the 2-year survival rate is reportedly only about 40% [22,24]. Therefore, the development of new curative therapies is required.

To prevent the onset of ATL, the elimination of HTLV-1-infected cells is considered important, but there are currently no means by which to accomplish this goal. To address this gap, we have been working on the development of virotherapy, a therapeutic method that targets, lyses, and kills HTLV-1-infected cells by using a cytolytic recombinant vesicular stomatitis virus (rVSV) and recombinants that express the HTLV-1 receptor [25]. VSV is a prototype rhabdovirus with a non-segmented, negative-stranded RNA genome that encodes five structural proteins (N, P, M, G, and L). VSV G is the only envelope glycoprotein; this protein attaches the virus to a host cell surface receptor and catalyzes pH-dependent viral entry into the cell. VSV infects various mammalian cell types in vitro, after which it replicates, producing numerous progeny virions, and rapidly induces cytolysis in infected cells [26,27,28]. In contrast, HTLV-1 employs glucose transporter 1 (GLUT1), neuropilin 1, and heparan sulfate proteoglycans, including syndecan 1, as receptors to enter host cells [29,30,31]. The HTLV-1 genome encodes *gag*, *pro*, *pol*, *env*, and *pX* [32], and the *env* gene codes for envelope glycoproteins (Env, gp46, and gp21) that are responsible for the specific binding of HTLV-1 to cellular receptor(s) [33]. Previously, we found that an rVSV engineered to express a primary HTLV-1 receptor was effective at targeting HTLV-1-infected Env-expressing cells under in vitro culture conditions and in an HTLV-1-infected humanized mouse model [25]. In particular, in HTLV-1-infected humanized mice, it was found that rVSV expressing the HTLV-1 receptor was capable of efficiently preventing HTLV-1-induced leukocytosis in the periphery and eliminating HTLV-1-infected Env-expressing cells in the lymphoid tissues. However, further preclinical studies are required for the practical application of this novel anti-HTLV-1 virotherapy.

Simian T-cell leukemia virus (STLV), a known counterpart of HTLV, is prevalent among various non-human primates in Asia and Africa but not in America [34,35]. STLV-1 is closely related genetically to HTLV-1 [36,37,38], and thirty-one Old World non-human primate (NHP) species have been reported as being naturally infected with STLV-1 [35]. Japanese macaques (JMs; *Macaca fuscata*) are among the NHP species that can be infected with STLV-1, and the reported sequence homology of the JM STLV-1 genome to that of HTLV-1 is 90% [38]. In addition, as with HTLV-1 infection, STLV-1 infection occurs mostly in the CD4^+^ T cells, although STLV-1 Tax expression has also been detected in bone marrow hematopoietic stem cells in vivo, and viral DNA was retrieved in all myeloid and lymphoid cells derived from STLV-1-infected progenitors [35,39]. Notably, the STLV-1 proviral loads and anti-STLV-1 antibody titers in JMs are mostly comparable with those of HTLV-1-infected humans [40]. Thus, STLV-1-infected JMs have similar characteristics to individuals infected with HTLV-1, and they are considered to be useful as an animal model for HTLV-1 infection. Therefore, we performed in vitro verification to assess if the drug evaluation of rVSV in an STLV-1-infected NHP model is suitable as a preclinical study.

In this study, we determined whether the newly constructed rVSVs carrying an STLV-1 primary receptor specifically recognize and infect STLV-1 Env-expressing cells and induce cytolysis in the infected cells in vitro.

## 2. Materials and Methods

### 2.1. Cells

BHK-21 cells (Riken BRC Cell Bank, Tsukuba, Japan) and Lenti-X 293T cells (Takara Bio, Shiga, Japan) were cultured in Dulbecco’s modified Eagle’s medium (DMEM) (Sigma-Aldrich, St. Louis, MO, USA) supplemented with 10% fetal bovine serum (FBS) and 1% antibiotic-antimycotic (Gibco, Carlsbad, CA, USA). BHK-G cells were cultured in DMEM supplemented with 10% FBS, 750 mg/mL geneticin, and 0.5 mg/mL tetracycline in accordance with previous studies [41,42]. BHK-G cells were induced to express VSV G under tetracycline-free culture conditions [41,42].

### 2.2. Construction of Plasmids

To construct rVSV plasmids lacking the G gene and encoding AcGFP1 and/or Japanese macaque GLUT1 (JM GLUT1), genes encoding these proteins were initially amplified by PCR with the expression vector for AcGFP1 (purchased from Clontech, Mountain View, CA, USA) or the cDNA derived from Si-2 cells, which are an STLV-1-carrying Japanese macaque lymphoid cell line. All rVSV plasmids were generated in accordance with standard recombination techniques as described previously [27,41]. Briefly, to generate plasmids that yield AcGFP1 or JM GLUT1 expression, rVSV G-deleted pVSV-XN2 (a VSV Indiana wildtype vector) was digested with restriction enzymes MluI and NheI (New England BioLabs, Ipswich, MA, USA), and the amplification products of AcGFP1 and JM GLUT1 were combined in a Gibson Assembly reaction using the Gibson Assembly Master Mix (New England BioLabs, Ipswich, MA, USA); these products were designated as pVSVΔG-AcGFP and pVSVΔG-JmGL, respectively. Subsequently, to generate a plasmid that yields both AcGFP1 and JM GLUT1 expression, pVSVΔG-AcGFP was digested with the restriction enzymes MluI and XhoI, and an amplification product of JM GLUT1 was combined in a Gibson Assembly reaction using Gibson Assembly Master Mix and designated as pVSVΔG-JmGL-AcGFP. The primer sequences used for these amplifications are listed in Table S1.

STLV-1 Env expression plasmids containing the *env* gene sequence derived from Si-2 cells or STLV-1-infected Japanese macaque-peripheral blood mononuclear cells (JM PBMCs) were prepared by the GENEWIZ in South Plainfield, NJ, USA. These plasmids were designated as pCAGGS-Si-2-Env or pCAGGS-JM-Env, respectively.

### 2.3. Recoveries of rVSVs

The rVSVs expressing an STLV-1 receptor molecule and complemented or not with AcGFP1 without the G protein were recovered from the pVSVΔG-JmGL, pVSVΔG-JmGL-AcGFP, and pVSVΔG-AcGFP plasmid vectors of rVSV by methods slightly modified from those described previously [25,27,41]. Briefly, BHK-G cells were plated and grown to approximately 50% confluency on 100-mm-diameter dishes under tetracycline-free culture conditions. The cells were then infected with vTF7-3, a recombinant vaccinia virus, at a multiplicity of infection (MOI) of 10 to induce T7 RNA polymerase. One hour later, these cells were transfected with the rVSV plasmid vectors and helper plasmids (pBS-N, pBS-P, pBS-L, and pBS-G) using Polyethylenimine (PEI) Max (Polysciences, Warrington, PA, USA) and then incubated at 37 °C for 48 h. The culture supernatants were subsequently passed through a 0.2-µm-pore sized filter to remove the majority of the vaccinia virus and transferred to fresh BHK-G cells previously induced to express G protein. After 48 h, culture supernatants were passed through a 0.1-µm-pore sized filter to completely remove the residual vaccinia virus and then were added to BHK-G cells expressing the G protein again. The recovery of infectious viruses was confirmed by microscopy of the VSV-mediated cytopathic effect and/or GFP expression. Individual plaques were isolated and grown on BHK-G cells previously induced to express the G protein. Culture supernatants containing such G-complemented viruses were then harvested and stored at −80 °C until use.

### 2.4. Preparation of Non-G-Complemented rVSV Stocks

To generate viruses that were not complemented with VSV G, G-complemented rVSVs were used to infect 80% confluent BHK-21 cells at an MOI of approximately 1. After adsorption of the viruses at 37 °C for 3 h, the supernatants were removed, and the cells were washed five times with FBS-free DMEM to remove residual input G-complemented viruses. The cells were then incubated in a fresh culture medium at 37 °C for 48 h, after which the supernatants were clarified by centrifugation and used as stocks for non-G-complemented viruses. The non-G-complemented viruses were incubated with anti-VSV G neutralizing antibodies I1 and I14 (at 1:100 dilution of each) (Kerafast, Boston, MA, USA) at 37 °C for 30 min immediately before the use of an aliquot of each virus to minimize the chances of any infection mediated by residual G protein.

### 2.5. Immunofluorescence Test

To assess the surface expression of JM GLUT1 encoded by the VSV genome, 40,000 BHK-21 cells were seeded into each well of a 12-well culture plate, and the following day, the cells were infected with G-complemented VSVΔG-JmGL (MOI of 0.1). At 1 day post-infection, the cells were stained with mouse monoclonal anti-Human GLUT1 antibody (Ab) (R&D Systems, Minneapolis, MN, USA), followed by incubation with a FITC-conjugated goat anti-mouse IgG (H + L) Ab (Jackson ImmunoResearch, West Grove, PA, USA) in accordance with the manufacturer’s recommendations. The stained cells were observed and photographed with an EVOS FL Auto Imaging System (Thermo Fisher Scientific, Waltham, MA, USA). To assess the infectivity and specificity of rVSVs, 20,000 BHK-21 cells were seeded into each well of a 24-well culture plate, and the following day, the cells were transfected with a pCAG empty vector or STLV-1 Env expression plasmid as described above using the PEI Max in accordance with the manufacturer’s protocol. After incubation for 24 h, these cells were inoculated with an equal volume (0.25 mL of viral stock) of non-G-complemented rVSVs. Additionally, non-G-complemented rVSVs (0.25 mL each), including VSVΔG-JmGL, VSVΔG-JmGL-AcGFP, and VSVΔG-huGL encoding human-GLUT1 gene [25] were pre-incubated with or without the HTLV-1 Env-neutralizing rat monoclonal antibody LAT-27 [43,44] (10 µg/mL) at 37 °C for 1 h; additionally, the cells transfected with the STLV-1 Env expression plasmid were pre-incubated with or without LAT-27 under the same conditions. The pre-incubated viruses were then inoculated onto those cells. At 48 h post-VSV infection, rVSV-infected cells were assessed by observing their GFP expression, the staining of GLUT1 was performed as described above, or the intracellular staining of VSV N protein was conducted as described previously [25,41]. The cells were photographed with an EVOS FL Auto Imaging System, and the total number of fluorescent cells in each well visible via fluorescence microscopy was counted to calculate the relative infectivity of rVSV.

### 2.6. Titration of rVSVs

The titers of rVSVs were measured by a method slightly modified from those described previously [25,41]. In brief, for the titration of G-complemented VSVs, viral stocks were serially diluted with FBS-free DMEM, and then the diluted samples were inoculated onto BHK-21 cells. For the titration of non-G-complemented VSVs, aliquots of viral stocks (0.25 mL each) were inoculated by neutralizing anti-VSV G monoclonal antibodies I1 and I14 onto BHK-21 cells that had been previously transfected with an expression plasmid for STLV-1 Env. At 12 h post-VSV inoculation, the cells were fixed and, if necessary, stained for VSV N protein, and the total number of fluorescent cells was counted as described above. The titer of each rVSV was calculated and expressed as the number of infectious units (IU) per milliliter.

### 2.7. Flow Cytometric Analysis

The flow cytometry analysis of STLV-1 Env expression was performed by an established method as described previously [25]. In brief, cells were pretreated with FcR blocking reagent (Miltenyi Biotec, Bergisch Gladbach, Germany). For the staining of HTLV-1 Env, mouse monoclonal anti-HTLV-1 gp46 Ab [67/5.5.13.1] (Abcam, Cambridge, United Kingdom) [25], mouse IgG1 kappa monoclonal Ab (isotype) (Abcam, Cambridge, United Kingdom), and goat anti-mouse IgG-PE Ab (Abcam, Cambridge, United Kingdom) were used in accordance with the manufacturer’s protocol. After staining, a flow cytometric analysis was immediately performed on a BD Accuri C6 flow cytometer with CFlow software (Becton Dickinson, Franklin Lakes, NJ, USA), and the collected data were analyzed by FCS Express 4 (De Novo Software, Los Angeles, CA, USA).

### 2.8. Cell-Killing Assay

To evaluate the killing effect of rVSVs on STLV-1 Env-expressing or -non-expressing cells, 1 × 10^5^ Lenti-X 293T cells were seeded into each well of 24-well culture plates, and the following day, the cells were transfected with a pCAG empty vector or pCAGGS-Si-2-Env using the PEI Max. After incubation for 24 h, these cells were inoculated with G-complemented rVSVs, including VSVΔG-JmGL, VSVΔG-JmGL-AcGFP, and VSVΔG-AcGFP at an MOI of 1. The cell viability was determined by applying the standard trypan blue staining method with a Countess automated cell counter (Thermo Fisher Scientific, Waltham, MA, USA) in accordance with the manufacturer’s instructions.

### 2.9. Statistical Analysis

Statistical analysis was performed using GraphPad Prism 9 (GraphPad Software, La Jolla, CA, USA). The significance of differences was determined by Student’s *t*-tests. *p*-values of <0.05 were considered statistically significant.

## 3. Results

### 3.1. Construction and Generation of VSV Recombinants Expressing STLV-1 Receptor

GLUT1 plays a key role in glucose transport [45] and is a major receptor for HTLV-1 [31,46]. We cloned the *Macaca fuscata* GLUT1 (Japanese macaque [JM] GLUT1) gene from Si-2 cells, which were obtained from the Japanese Collection of Research Bioresources Cell Bank (JCRB1321) and belong to an STLV-1-infected Japanese macaque cell line, and identified the amino acid sequence (accession No. LC684847). The resulting data revealed that JM GLUT1 shares 99% amino acid sequence identity with human GLUT1 (Table 1). The amino acid sequences of extracellular loop 5 (ECL5) and ECL6, which are involved in Env binding and fusion events [47], are completely identical, whereas the two amino acids in ECL1 that are involved in viral entry and the determination of viral tropism [46,47] were different between these sequences (Table 1). Notably, the amino acid sequences of the GLUT1 binding site in HTLV-1 Env and STLV-1 Env were found to be completely identical (data not shown).

**Figure 1:**
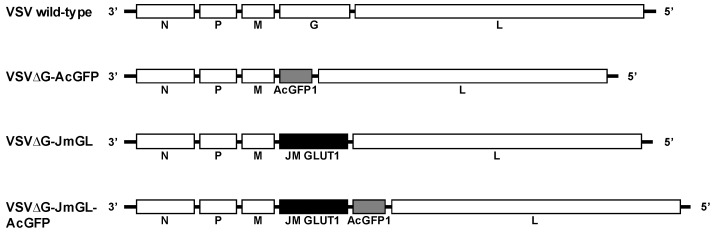
Schematic showing the structure of rVSVs expressing an STLV-1 receptor molecule and AcGFP1 as a reporter protein. The gene orders in the wildtype VSV and the rVSV constructs used in this study are illustrated. The deleted G gene was replaced with the JM GLUT1 or AcGFP1 genes, yielding the VSVΔG-JmGL or VSVΔG-AcGFP constructs, respectively. In addition, a JM GLUT1 gene was inserted upstream of the AcGFP1 gene in VSVΔG-AcGFP, yielding VSVΔG-JmGL-AcGFP.

Here, to develop a preclinical evaluation system that could be tested against STLV-1 in a non-human primate model, we generated recombinant VSV expressing JM GLUT1 for targeting cells expressing the STLV-1 Env protein. The G gene was deleted from the wildtype VSV (Indiana strain) plasmid construct (pVSV-XN2), and the JM GLUT1 gene was inserted between the M and L genes as a substitute for the deleted G gene (Figure 1). The virus recovered from this construct was designated as VSVΔG-JmGL. Next, we generated recombinant VSV expressing the AcGFP1 gene, designated as VSVΔG-AcGFP, by applying the same approach that we used to generate VSVΔG-JmGL, and this virus was used as a negative control (Figure 1). In addition, the gene encoding JM GLUT1 was inserted upstream of the AcGFP1 gene in VSVΔG-AcGFP in an attempt to trace the virus infection in vitro. The virus recovered from this construct was designated as VSVΔG-JmGL-AcGFP (Figure 1). Because of the deletion of the VSV G gene, the viruses were initially recovered in the presence of complementing G protein and were subsequently propagated in BHK-21 cells expressing VSV G protein [42].

### 3.2. Cell Surface Expression of the STLV-1 Receptor Molecule Encoded in the rVSV Genome

**Figure 2:**
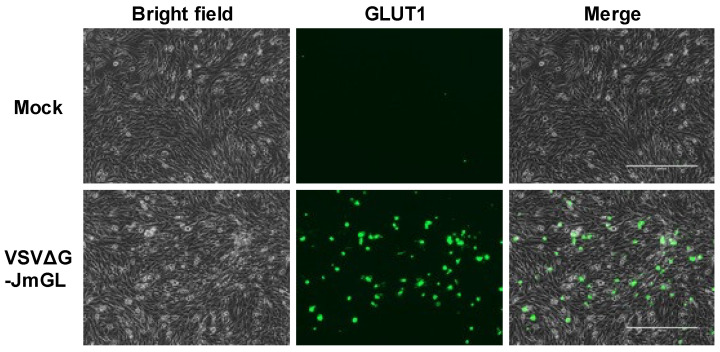
Expression of the STLV-1 receptor molecule on the rVSV-infected target cell surface. The surface expression of JM GLUT1 protein after VSV infection was confirmed by immunofluorescence staining. VSV-permissive BHK-21 cells were infected with G-complemented VSVΔG-JmGL at an MOI of 0.1. After 1 day of culture, JM GLUT1 expressed by the viral genome was stained with anti-GLUT1-specific mouse monoclonal antibody (MAb) followed by FITC-conjugated anti-mouse IgG. The stained cells were observed by fluorescence microscopy and photographed at a constant magnification. Scale bars in panels represent 400 µm.

To evaluate whether rVSV expressed the STLV-1 receptor molecule encoded by the viral genome, we examined the protein expression of the receptor on the surface of infected cells by immunofluorescence staining. We initially infected BHK-21 cells with G-complemented VSVΔG-JmGL at an MOI of 0.1, and the cell surface expression of JM GLUT1 was evaluated by immunofluorescence microscopy. As shown in Figure 2, the expression of GLUT1 protein was observed in the VSVΔG-JmGL-infected BHK-21 cells, and the result suggests the receptor molecule was successfully expressed from the encoding viral genome, transported to the cell surface, and presumably incorporated into the progeny virions in rVSV-infected cells.

### 3.3. Specific Infection of STLV-1 Env-Expressing Cells by rVSV Carrying the JM GLUT1

**Figure 3:**
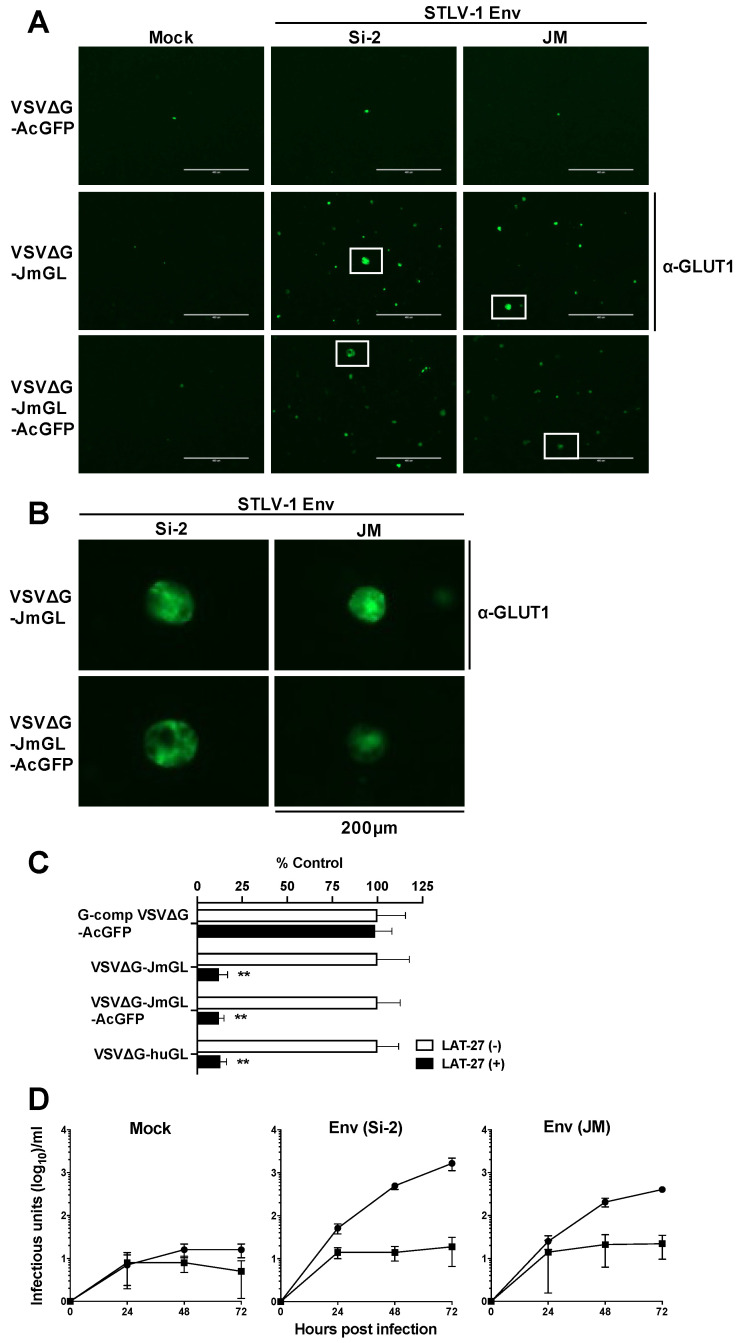
Functional assessment of infectivity and specificity of rVSVs. (**A**,**B**) STLV-1 Env-dependent infectivity of rVSVs was evaluated. BHK-21 cells were initially mock-transfected (empty vector) or transfected with expression plasmids for STLV-1 Env derived from Si-2 cells or from STLV-1-infected JM PBMCs. After 24 h, transfected BHK-21 cells were infected with non-G-complemented rVSVs (0.25 mL each). After 3 days of culture, the cells were fixed. Subsequently, VSVΔG-JmGL-infected cells were stained with anti-GLUT1-specific MAb to detect rVSV-infected cells. On the other hand, VSVΔG-JmGL-AcGFP- or VSVΔG-AcGFP-infected cells were detected by GFP expression. Representative results from each group of rVSV-infected BHK-21 cells are shown in panel (**A**). Areas enclosed with squares are enlarged in panel (**B**). Syncytia with enlarged cell size in VSVΔG-JmGL- or VSVΔG-JmGL-AcGFP-infected cells are shown in panel (**B**). Scale bars in panels (**A**,**B**) represent 400 µm and 200 µm, respectively. (**C**) STLV-1 Env-dependent rVSV infection was assessed by neutralizing assay. BHK-21 cells were initially transfected with the Si-2 strain STLV-1 Env expression plasmid. After 24 h, transfected BHK-21 cells were infected with G-complemented VSVΔG-AcGFP (MOI of 0.01) or non-G-complemented rVSVs (0.25 mL each), in the absence or presence of HTLV-1 envelope glycoprotein-specific neutralizing antibody (LAT-27; 10 µg/mL). The cells infected with VSVΔG-JmGL or VSVΔG-huGL were stained and examined by VSV N-specific immunofluorescence to detect the rVSV-infected cells at 48 h post-infection. The culture of each virus without LAT-27 was defined as a control. The total number of fluorescent cells per well was determined, and the relative infectivity of each virus was calculated. The data are expressed as a percentage of the control (mean ± SD) from four independent experiments. (**D**) Viral growth kinetic assay results are shown. Si-2 strain STLV-1 Env-transfected BHK-21 cells were infected with non-G-complemented VSVΔG-AcGFP (square) or VSVΔG-JmGL-AcGFP (circle) (0.25 mL each). The total number of GFP-positive cells per well was counted to determine the quantitative viral titer (in infectious units per milliliter). The data are expressed as the mean ± SD from four independent experiments. Asterisks in panel (**C**) represent significant differences versus control (** *p* < 0.001 significance, by two-tailed Student’s *t*-test with equal variance).

As the direct detection of the incorporated receptor molecule on VSV particles was not made, we performed subsequent functional experiments to confirm that JM GLUT1 was incorporated into the VSV particles. To determine the infectivity of rVSV carrying the JM GLUT1, we conducted rVSV infection experiments using target cells expressing two types of STLV-1 Env. Initially, we constructed expression plasmids for STLV-1 Env derived from Si-2 cells (Si-2 strain) and STLV-1-infected JM PBMCs (JM strain). These two Env proteins have the same GLUT1-binding site but differ in 6 of their 488 amino acids (data not shown). Subsequently, we mock-transfected (empty vector) or transfected BHK-21 cells with STLV-1 Env expression plasmids, and then infected these cells with VSVΔG-JmGL or VSVΔG-JmGL-AcGFP, which were not complemented with the VSV G protein (non-G-complemented viruses). Non-G-complemented VSVΔG-AcGFP was used as an experimental control. At 3 days post-infection (dpi) under culture conditions in the presence of G protein-neutralizing antibodies, VSVΔG-JmGL-AcGFP- or VSVΔG-AcGFP-infected cells were detected by GFP expression because these rVSVs carry the AcGFP gene. On the other hand, VSVΔG-JmGL-infected cells were detected by rVSV-induced GLUT1-specific immunofluorescence staining, but not by GFP expression, because VSVΔG-JmGL does not carry the AcGFP gene. As shown in Figure 3A, non-G-complemented VSVΔG-JmGL and VSVΔG-JmGL-AcGFP each successfully infected the cells expressing STLV-1 Env derived from either Si-2 or JM strain, but they did not infect mock-transfected (non-Env-expressing) cells (Figure 3A). In contrast, as anticipated, VSVΔG-AcGFP did not infect cells in the presence or absence of STLV-1 Env protein expression.

We also observed larger cells among the rVSV-infected cells (Figure 3A,B). We previously reported that virus receptor molecules expressed on the surface of rVSV-infected cells mediate cell–cell fusion if the cells also express a specific Env protein, such as HTLV-1 Env [25,41,48,49]. Accordingly, infection by either VSVΔG-JmGL or VSVΔG-JmGL-AcGFP resulted in cell fusion (syncytium formation) in both cells expressing the Si-2 strain STLV-1 Env and cells expressing the JM strain STLV-1 Env (Figure 3A,B). These results indicate that functional JM GLUT1 was expressed by cells infected with either VSVΔG-JmGL or VSVΔG-JmGL-AcGFP.

Next, to confirm that rVSVs carrying the JM GLUT1 could specifically infect STLV-1 Env-expressing target cells, we examined the specificity of rVSV infection by conducting a neutralizing assay. We used the HTLV-1 Env-specific neutralizing antibody LAT-27 as a neutralizing antibody against STLV-1 Env in this study because STLV-1 Env has the same LAT-27 recognition sequence contained in HTLV-1 Env [44]. As demonstrated above, infectious non-G-complemented rVSVs, such as VSVΔG-JmGL and VSVΔG-JmGL-AcGFP, were pretreated with or without LAT-27. In addition, non-G complemented VSVΔG-huGL, which carries the human GLUT1 gene, was used as a positive control [25], and G-complemented VSVΔG-AcGFP was used as a control of G-mediated infection. At 48 h post-infection, cells infected with VSVΔG-JmGL or VSVΔG-huGL were detected by VSV-N-specific immunofluorescence staining, and cells infected with VSVΔG-JmGL-AcGFP or VSVΔG-AcGFP were also detected by GFP expression. Fluorescence microscopy results revealed that the infectivity of non-G-complemented VSVΔG-JmGL and VSVΔG-JmGL-AcGFP was significantly reduced by treatment with LAT-27 (80–90% reduction) compared with mock-treated viruses, as observed for non-G-complemented VSVΔG-huGL (Figure 3C). In contrast, G-complemented VSVΔG-AcGFP infection was not affected by treatment with LAT-27 (Figure 3C). These results indicate that the infectivity of non-G-complemented VSVΔG-JmGL and VSVΔG-JmGL-AcGFP is specifically mediated by the interaction between STLV-1 Env at the cell surface and JM GLUT1 incorporated into the envelopes of VSV particles, but not by the G protein. Additionally, these results also show that VSVΔG-JmGL and VSVΔG-JmGL-AcGFP have similar characteristics and that AcGFP1 expression does not affect the infectivity or specificity of the virus.

Furthermore, we performed growth kinetic assays to assess the replicative abilities of rVSVs in STLV-1 Env-expressing cells. As shown in Figure 3D, non-G-complemented VSVΔG-JmGL-AcGFP underwent replication in both the cells expressing Si-2 strain STLV-1 Env and those expressing JM strain STLV-1 Env during the culture period, whereas non-G-complemented VSVΔG-AcGFP barely replicated in these cells. Although non-G-complemented VSVΔG-AcGFP did not establish an infection in the cells (Figure 3A), it is likely that the virus transiently infected them and grew via residual G complementation, even when the virus was treated with anti-VSV neutralizing antibodies before being used for inoculation (Figure 3D). The data confirm that the VSVΔG-JmGL-AcGFP is appropriate as a candidate for use in a preclinical study for further experimentation.

### 3.4. Elimination of STLV-1 Env-Expressing Cells by Infection with VSVΔG-JmGL-AcGFP

**Figure 4:**
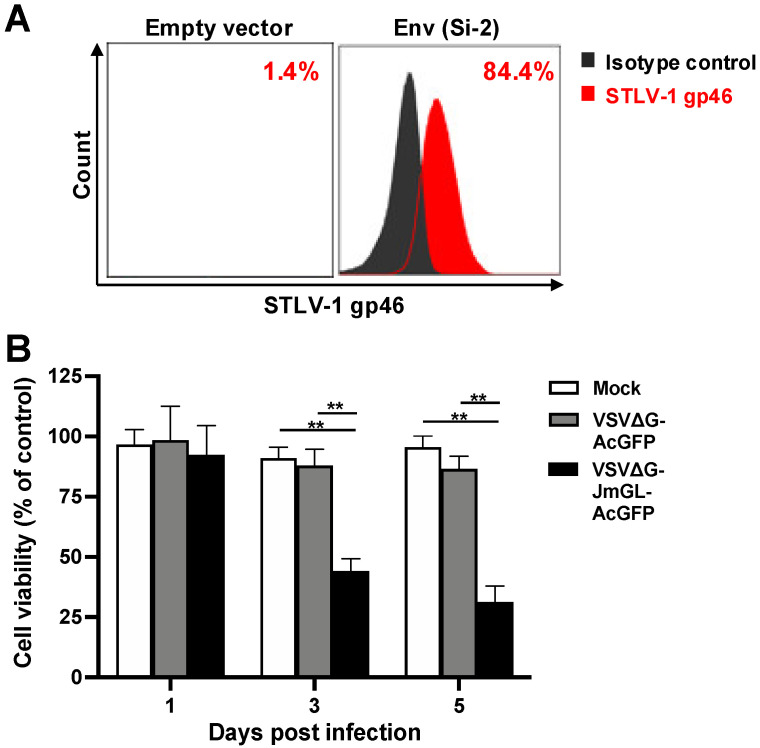
Reduction of STLV-1 Env-expressing target cells by infection with rVSV. (**A**) The expression level of STLV-1 Env by Lenti-X 293T cells was analyzed at 24 h after their transfection with an empty vector as a control or STLV-1 Env (Si-2 strain) expression plasmid. These cells were stained with anti-HTLV-1 gp46 MAb or mouse IgG1 MAb as an isotype control and then subjected to flow cytometry. The percentages of STLV-1 gp46-positive cells are presented in each panel. (**B**) Results from an evaluation of the rVSV-mediated killing effects on STLV-1-Env expressing cells are shown. The cells described in (**A**) were mock-infected or infected with G-complemented VSVΔG-AcGFP or VSVΔG-JmGL-AcGFP at an MOI of 1. The numbers of viable cells were counted on days 1, 3, and 5 after infection, and the cell viabilities (% of control) of each group of rVSV-infected Si-2-Env-expressing cells relative to each group of virus-inoculated control cells were calculated. Data obtained from five independent experiments are expressed as the mean ± SD. Asterisks in panel (**B**) represent significant differences versus mock- or VSVΔG-AcGFP-infected cells (** *p* < 0.001 significance, by two-tailed Student’s *t*-test with equal variance).

We previously demonstrated that rVSVs encoding receptor molecules for a target virus specifically superinfect and kill target virus-infected cells [25,41,48,49]. Therefore, to examine whether similar cytolytic effects could be elicited by rVSV carrying JM GLUT1, we performed a cell-based killing assay targeting STLV-1 Env-expressing cells. First, we performed flow cytometry analysis to evaluate the STLV-1 Env expression on the cell surface after transfection with the STLV-1 Env expression plasmid. As expected, we found that the transfected 293T cells expressed STLV-1 Env on the cell surface (Figure 4A). Subsequently, cells transfected with an empty vector as a control or STLV-1 Env (Si-2 strain) were mock-infected or infected with G-complemented VSVΔG-JmGL-AcGFP or VSVΔG-AcGFP at an MOI of 1, and the numbers of viable cells were then counted at the indicated days after infection. Because our G-complemented rVSVs were more infectious (i.e., higher titer) than our non-G-complemented rVSVs, we used the G-complemented rVSVs in this experiment. The cell viabilities (% control) of each group of rVSV-infected STLV-1 Env-expressing cells relative to the corresponding virus-inoculated control cell groups are shown in Figure 4B. The cell viabilities of VSVΔG-JmGL-AcGFP-infected cells at 3 and 5 days after infection were 44.1 ± 5.0 and 31.3 ± 6.5, respectively, and the number of STLV-1 Env-expressing cells was dramatically reduced. In contrast, the cell viabilities of mock- or VSVΔG-AcGFP-infected cells at the same timepoint were 91.0 ± 4.5 and 95.6 ± 4.5 or 87.9 ± 6.8 and 86.5 ± 5.3, respectively. The results show that the cell viability in the VSVΔG-JmGL-AcGFP-infected cells was significantly lower compared with that of mock- or VSVΔG-AcGFP-infected cells at 3 and 5 days after infection (*p* < 0.001). These results suggest that STLV-1 Env-expressing cell lines are susceptible to rVSV infection when expressing JM GLUT1, which is consistent with the results described in Figure 3. We also confirmed that VSVΔG-JmGL-AcGFP has a greater potential killing activity via superinfection of STLV-1 Env-expressing cells compared with the other rVSV.

## 4. Discussion

In JMs naturally infected with STLV-1, STLV-1 Tax and SBZ function similarly to their counterpart proteins in HTLV-1, which indicates that STLV-1-infected JMs resemble asymptomatic HTLV-1 carriers [50]. On the basis of this discovery, STLV-1 infection in naturally infected JMs has been used as a model for the persistent HTLV-1 infection found in HTLV-1 carriers. The anti-CCR4 monoclonal antibody, mogamulizumab, which is currently employed for treating relapsed ATL, was found to also be effective against STLV-1 infection in asymptomatic JMs [50,51]. This finding suggests that STLV-1-infected JMs are an excellent model for evaluating anti-HTLV-1 drug candidates.

A number of recombinant viruses that specifically infect and kill malignant cells have been developed to treat several types of cancer; the therapeutic application of these recombinant viruses is known as virotherapy [52,53,54]. Their therapeutic effects are mediated by virus-induced oncolysis [55,56]. VSV is a reasonable choice for a virotherapy candidate because established rVSV vectors are robustly cytolytic and genetically tractable [57]. To establish a new mode of VSV-based virotherapy, we previously reported the development of rVSVs that can specifically superinfect and successfully kill HIV-1-, SIV-, or HTLV-1-infected cells, leading to infection control [25,41,48,49]. These recombinant viruses express single or multiple HIV-1/SIV/HTLV-1 receptors in place of the VSV G protein and eliminate only HIV-1/SIV/HTLV-1 Env-expressing target cells as they are dependent on specific interactions (i.e., binding and subsequent membrane fusion) between the receptors incorporated into the virion surface and the Env proteins expressed on the target cell surface. To further verify the therapeutic value of anti-HTLV-1 virotherapy with rVSVs, in the present study, we generated novel rVSVs for targeting STLV-1 Env-expressing cells, with the goal of applying them to a preclinical study performed with the HTLV-1 surrogate animal model, STLV-1-infected JMs.

Because human GLUT1 and NRP1 were identified as the primary HTLV-1 receptors, which are involved in HTLV-1 entry into host cells via HTLV-1 gp46 and gp21 Env glycoproteins [58,59,60], we initially tried to obtain clones of homologues for both GLUT1 and NRP1, i.e., simian (JM) GLUT1 and NRP1, respectively. However, only the JM GLUT1 gene could be successfully isolated from an STLV-1-infected JM cell line, Si-2 cells. Although the reason why we were unable to obtain a clone of the JM NRP1 gene is still unclear, we speculate that the expression of the JM NRP1 gene might be even more downregulated than that of the JM GLUT1 gene as a consequence of the STLV-1 infection of the cells. Thus, we constructed only rVSVs expressing JM GLUT1 for targeting STLV-1 Env-expressing cells via their interaction with Env. As observed for the other rVSVs that we generated in previous studies [25,41,48,49], in vitro infection with G-complemented VSVΔG-JmGL-AcGFP clearly reduced the number of STLV-1 Env-expressing cells, most likely through virus-mediated membrane fusion following receptor–Env binding, whereas infection with the control virus, G-complemented VSVΔG-AcGFP, did not (Figure 4B). It is reasonable to assume that the rVSVs would also use these mechanisms to target and eliminate STLV-1-infected Env-expressing cells in vivo. Moreover, although G complementation can elicit G-mediated infection of a broad range of cells, the effect of this mechanism was negligible here, as demonstrated by the case of G-complemented VSVΔG-AcGFP infection, because transient G complementation can enable rVSVs to potentially enter and kill unrelated normal cells only once.

Our rVSVs have several possible advantages for use in retrovirus-targeting virotherapy. (i) Because these rVSVs are replication-competent, their therapeutic potency can be sustained during infection. (ii) Because the replication and growth of these rVSVs are dependent on retroviral Env-expressing cells, the levels of rVSV replication will decline in parallel with retrovirus clearance, which contributes to the likely clinical safety of their usage. (iii) Because the viral receptors incorporated into the surface of these rVSVs are normal host proteins, they should not induce host production of neutralizing antibodies that inhibit viral spread. (iv) The release of internal viral antigens following the cytolysis produced by infection with rVSVs can induce cellular immune responses, such as cytotoxic T lymphocytes directed against VSV- and/or retrovirus-infected cells, which may induce a systemic immune response and enhance the elimination of target cells. This advantage suggests that rVSVs might be applicable as potent therapeutic vaccines [61,62,63]. (v) An escape mutation of retroviral Env against the rVSVs is unlikely to occur because this mutation would probably result in the loss of Env binding to native primary receptors on the cell surface, eventually leading to the loss of retroviral infectivity to susceptible host cells.

We previously found that the expression of HTLV-1 Env was restricted to cells in the bone marrow and spleen of infected humanized mice, and little was detected in the peripheral blood of infected mice [25]. Thus, the expression of HTLV-1 proviral genes is definitely activated in the hematopoietic organs, indicating that these organs have potential as therapeutic targets for our rVSV antiretroviral strategy, even if the viral expression is strongly suppressed in the periphery, as is observed in HTLV-1-infected individuals [64,65,66]. Similarly, it was previously reported that STLV-1 Tax expression is particularly high in the bone marrow of STLV-1-infected JMs, indicating that STLV-1 infects hematopoietic stem cells [39]; therefore, VSVΔG-JmGL or VSVΔG-JmGL-AcGFP might potentially target and eliminate STLV-1 Tax-inducible Env-expressing hematopoietic stem cells in STLV-1-infected JMs. On the basis of these findings, our preliminary study to investigate whether STLV-1 Tax expression is capable of inducing Env expression in STLV-1-infected peripheral blood mononuclear cells and/or splenocytes in STLV-1-infected JMs ex vivo is in progress. After confirming the Env expression, we would check whether the new rVSV can actually kill these JM STLV-1-infected Env-expressing primary cells and/or cell lines established in vitro.

In conclusion, we have generated novel rVSVs, termed VSVΔG-JmGL, with or without the AcGFP reporter gene, that target and eliminate STLV-1 Env-expressing cells in our HTLV-1 surrogate model in vitro. These antivirals can now be tested in STLV-1 naturally infected JMs, i.e., the non-human primate model that mimics HTLV-1 infection, to determine their therapeutic values in vivo. Our study strongly indicates that rVSVΔGs carrying an STLV-1/HTLV-1 receptor might be a candidate for unique virotherapy in STLV-1/HTLV-1-infected individuals, potentially leading to a prophylactic strategy against the development of HTLV-1 infection-related diseases.

## Figures and Tables

**Table 1:** Amino acid sequence identity between human GLUT1 and Japanese macaque GLUT1.

Species	Accession No.	Description	Identity (Percentage)	Different Amino Acid Positions			
				49	55	183	195
				Outside (ECL1)	Outside (ECL1)	Outside (ECL1)	TMhelix
*Homo sapiens*	NP_006507.2	Solute carrier family 2, facilitated glucose transporter member 1	-	V	S	K	I
*Macaca fuscata*	LC684847	Macaca fuscata GLUT-1 (from Si-2 cells)	488/492 (99%)	I	R	E	V

## Data Availability

The data presented in this study are available in the article or the Supplementary Material.

## Supplementary Materials

The following supporting information can be downloaded at: https://www.mdpi.com/article/10.3390/v14040740/s1, Table S1: Primers used for rVSV construction.
